# Lower Ankle-Brachial Index Is Related to Worse Cognitive Performance in Old Age

**DOI:** 10.1037/neu0000028

**Published:** 2013-12-02

**Authors:** Erika J. Laukka, John M. Starr, Ian J. Deary

**Affiliations:** 1 Aging Research Center, Department of Neurobiology, Care Sciences, and Society, Karolinska Institutet and Stockholm University, Stockholm, Sweden; 2 Centre for Cognitive Ageing and Cognitive Epidemiology and Geriatric Medicine Unit, University of Edinburgh, Edinburgh, United Kingdom; 3 Centre for Cognitive Ageing and Cognitive Epidemiology and Department of Psychology, University of Edinburgh, Edinburgh, United Kingdom

**Keywords:** cognition, ankle-brachial index, peripheral artery disease, atherosclerosis, aging

## Abstract

***Objective:*** We aimed to study the associations between peripheral artery disease (PAD) and ankle-brachial index (ABI) and performance in a range of cognitive domains in nondemented elderly persons. ***Methods:*** Data were collected within the Lothian Birth Cohort 1921 and 1936 studies. These are two narrow-age cohorts at age 87 (*n* = 170) and 73 (*n* = 748) years. ABI was analyzed as a dichotomous (PAD vs. no PAD) and a continuous measure. PAD was defined as having an ABI less than 0.90. Measures of nonverbal reasoning, verbal declarative memory, verbal fluency, working memory, and processing speed were administered. Both samples were screened for dementia. ***Results:*** We observed no significant differences in cognitive performance between persons with or without PAD. However, higher ABI was associated with better general cognition (β = .23, *p* = .02, *R*^2^ change = .05) and processing speed (β = .29, *p* < .01, *R*^2^ change = .08) in the older cohort and better processing speed (β = .12, *p* < .01, *R*^2^ change = .01) in the younger cohort. This was after controlling for age, sex, and childhood mental ability and excluding persons with abnormally high ABI (>1.40) and a history of cardiovascular or cerebrovascular disease. ***Conclusion:*** Lower ABI is associated with worse cognitive performance in old age, especially in the oldest old (>85 years), possibly because of long-term exposure to atherosclerotic disease. Interventions targeting PAD in persons free of manifest cardiovascular and cerebrovascular disease may reduce the incidence of cognitive impairment and dementia.

Old age is associated with high prevalence of vascular disease. In addition to being associated with adverse health outcomes and mortality, vascular disease is also associated with an increased risk of cognitive impairment. At one end of the spectrum, vascular disease and vascular risk factors are related to increased incidence of vascular dementia (Jellinger, 2008) and Alzheimer’s disease (de la Torre, 2009; Reitz et al., 2010). However, vascular cognitive impairment encompasses a range of cognitive disturbances, from dementia to subtle cognitive deficits with a vascular origin (Bowler, Steenhuis, & Hachinski, 1999; O’Brien, 2006), and constitutes a common cause of cognitive impairment in nondemented elderly persons (Rockwood et al., 2000). Furthermore, persons with vascular cognitive impairment show increased risk of deteriorating on different cognitive and functional measures compared with persons with no cognitive impairment (Rockwood et al., 2007).

A highly prevalent vascular condition in the older population is lower-extremity peripheral artery disease (PAD), causing narrowing of the peripheral arteries. The prevalence of PAD is strongly related to age, and PAD affects approximately 10% of the population by the age of 65 and over 20% after the age of 80 (Aboyans & Criqui, 2009). PAD is most easily detected through the measurement of the ankle-brachial index (ABI), which is obtained by dividing the systolic blood pressure in the ankle to that in the arm. The ABI is frequently used as a measure of generalized atherosclerosis, in which an ABI less than 0.90 is considered a sign of PAD and a lower ABI indicates increasing severity (Fowkes, 1988). PAD has been related to higher mortality, especially from cardiovascular disease (Criqui et al., 1992; Fowkes et al., 2008). It is important to note that higher mortality occurred also in persons who are traditionally not considered at increased risk of cardiovascular events, such as persons with a low or intermediate Framingham Risk Score (Fowkes et al., 2008; Murphy, Dhangana, Pencina, & D’Agostino, 2012). Similar elevated mortality rates have been reported for persons with abnormally high ABI (>1.40; Resnick et al., 2004).

A recent systematic review targeted the ABI as a marker of cognitive impairment in the general population (Guerchet et al., 2011). The authors concluded that a low ABI was associated with cognitive impairment, dementia, and future cognitive decline. In most studies, cognitive functioning was assessed with the Mini-Mental State Examination (MMSE; Folstein, Folstein, & McHugh, 1975), a common screening instrument for dementia. Few population-based studies targeting the ABI have included neuropsychological testing. In one, the Edinburgh Artery Study, a low ABI at baseline was associated with lower scores on Raven’s Matrices (nonverbal reasoning), Digit Symbol (processing speed), and verbal fluency 10 years later (Price et al., 2006). The strongest association was observed for processing speed. However, the ABI was not associated with change in cognitive performance over 5 years (Johnson, Price, Rafnsson, Deary, & Fowkes, 2010). In another study, a low ABI (<0.90) was associated with faster decline on Digit Symbol over 7 years (Haan, Shemanski, Jagust, Manolio, & Kuller, 1999), and PAD has been associated with significantly lower Digit Symbol performance in persons with microalbuminuria (Vupputuri, Shoman, Hogan, & Kshirsagar, 2008). Poorer cognitive performance has also been reported from studies defining PAD as the presence of intermittent claudication (Rafnsson, Deary, Smith, Whiteman, & Fowkes, 2007; Waldstein et al., 2003). However, large-scale studies on the association between ABI and cognition are scarce.

Another major limitation of previous studies is the lack of any premorbid measures of cognitive ability. This is important because lower cognitive ability in childhood is associated with a range of adverse vascular outcomes, such as hypertension (Starr et al., 2004), cardiovascular disease (Hart et al., 2004), and vascular dementia (McGurn, Deary, & Starr, 2008). Because a considerable proportion of the variance in mental ability in later life is explained by childhood mental ability (Deary, Whalley, Lemmon, Crawford, & Starr, 2000), associations between cognition and ABI in later life might be a consequence of the influence of childhood mental ability on both of these outcomes rather than representing any causal relationship.

The aim of the present study was to examine the associations between PAD and ABI and cognitive performance in a range of cognitive domains. On the basis of previous research, we hypothesized that higher ABI would be related to better cognitive performance. This was tested in two age-homogenous population-based samples of different ages, 87 and 73 years old, in which premorbid mental ability scores were available.

## Methods

### Participants

Data were collected within the Lothian Birth Cohort (LBC) 1921 and 1936 studies. Recruitment and data collection in these studies have been described in detail elsewhere (Deary, Gow, Pattie, & Starr, 2012; Deary et al., 2007; Deary, Whiteman, Starr, Whalley, & Fox, 2004b). In brief, these data collections follow up older people residing in the Edinburgh area of Scotland who participated in the Scottish Mental Surveys of 1932 and 1947 (Scottish Council for Research in Education, 1933, 1949). The participants were tested with a general intelligence test at age 11 and were later recruited for follow-up studies at mean ages of 79 (LBC1921) and 70 (LBC1936), respectively.

Of the 550 participants tested at wave 1 of the LBC1921, 321 persons (mean age = 83 years) came back for a second and 237 persons (mean age = 87 years) came back for a third wave of testing at approximately 4-year intervals. At wave 1 of the LBC1936, 1,091 participants were assessed, 866 (mean age = 73 years) of who came back for a second wave approximately 3 years later. Reasons for attrition were death, severe acute illness, refusal, and loss of contact. Each assessment involved an interview, cognitive testing, physical examination, and self-report questionnaires. Ethics permissions were obtained from the Multi-Centre Research and the Lothian Research Ethics Committees for Scotland, and the ethical guidelines from the World Medical Association Declaration of Helsinki were followed throughout all parts of the studies. Informed consent was collected from all participants.

For the present study, we used data from those data collection waves when a measurement of ABI was introduced. At wave 3 of the LBC1921, 207 persons participated in the cognitive testing. ABI was not assessed for 28 of these because the person was tested at home or because the measurement was disrupted because of discomfort for the participant. For the purpose of the present study, nine additional persons were excluded because of a low MMSE score (<24) or other indication of dementia in the medical history, resulting in a sample of 170 persons for the LBC1921.

At wave 2 of the LBC1936, 756 persons had data on ABI and cognition. Of these, eight persons were excluded because of low MMSE score or other indication of dementia, resulting in a sample of 748 persons for the LBC1936.

### Cognitive Assessment

For both cohorts, childhood mental ability was assessed with the Moray House Test No. 12. This is a general mental ability test that was validated against the Terman–Merill revision of the Binet scales (Scottish Council for Research in Education, 1933; 1949); thus, it could be used to calculate the persons’ IQ scores at age 11. The cognitive tests used at follow-up were largely overlapping for the two cohorts.

#### Nonverbal reasoning

Reasoning was measured by Raven’s Standard Progressive Matrices (LBC1921) or the Wechsler Matrix Reasoning subtest (LBC1936). Raven’s Matrices (Raven, Court, & Raven, 1977) is a 60-item test in which the participants are asked to choose the correct item to complete an incomplete pattern. Wechsler Matrix Reasoning is a subtest of the Wechsler Adult Intelligence Scale-III^U.K.^ (WAIS-III; Wechsler, 1998a). In this test, the participants are asked to examine a pattern arrayed in a matrix with one piece missing and choose the correct piece from the provided answer options.

#### Verbal declarative memory

In the Logical Memory subtest of the Wechsler Memory Scale (WMS; Wechsler, 1987, 1998b), the participants listen to two short stories. Immediately after each reading, and after a minimum of a 30-min delay, the participants are asked to tell the interviewer as much as they can remember from the stories. The score used was the total number of memory elements recalled from immediate and delayed recall for the two stories combined.

#### Verbal fluency

In letter fluency (Lezak, 2004), the participants are asked to name as many words as possible beginning with the letters C, F, and L, respectively, during 1 min. Proper names or repeated words are not credited. The score used was the total number of words generated across the three tasks.

#### Working memory

In Letter-Number Sequencing from the WAIS-III, the interviewer reads increasingly long strings of alternating numbers and letters. The participants are instructed to repeat first the numbers, in numerical order, and then the letters in alphabetical order. The score used was the number of correctly reproduced sequences.

#### Processing speed

In Digit Symbol (WAIS-III), the participants are instructed to enter symbols according to a given number-symbol code. The score recorded is the number of completed symbols within 2 min. Two reaction time (RT) tasks were administered, both with an interstimulus interval that varied between 1 and 3 s (Deary, Der, & Ford, 2001). In simple RT, there are 8 practice trials and 20 test trials. The participants are instructed to press the 0 key as fast as possible each time a 0 appears on a LCD screen. The mean RT of the 20 trials is calculated. The four-choice RT test has 8 practice trials and 40 test trials. When a number appears on the screen, the participants are to press the appropriate key (1, 2, 3, or 4) as quickly as possible. The score used was mean RT for correct trials.

For the LBC1921, the Moray House Test was readministered at age 87 using the same instructions and 45-min time limit as at age 11. The scores were corrected for age at time of testing and converted to standard IQ type scores (*M* = 100, *SD* = 15). A *g* factor score, representing general cognitive ability, was derived from three of the tests described above: Raven’s Matrices, Logical Memory-total, and letter fluency. For the LBC1936, a more comprehensive *g* factor score was derived from scores on six WAIS-III subtests: Letter-Number Sequencing, Matrix Reasoning, Block Design, Digit Symbol, Digit Span Backwards, and Symbol Search. Further, a *g* speed factor score was derived from scores on a set of processing speed measures: Symbol Search, Digit Symbol, simple and choice RT, and inspection time (a computer-based test of elementary visual processing speed; Deary et al., 2004a). A *g* memory factor score was derived from scores on a set of memory measures from WMS: Logical Memory I immediate and II delayed recall, Spatial Span forward and Spatial Span backward, Verbal Paired Associates I immediate and II delayed recall, and two WAIS-III subtests—Letter-Number Sequencing and Digit Span Backwards. The extraction of these factors, using principal component analysis, has been described in detail elsewhere (Corley et al., 2010; Luciano et al., 2009). In brief, regression scores were calculated for the first unrotated principal component for a *g* factor score. The same method was used to extract a *g* speed factor score and a *g* memory factor score from the tests listed above.

### Physical Examination and Interview

A physical examination was conducted at each wave by trained research nurses. Brachial systolic pressure was measured in the right arm after 5 min of rest using a Doppler ultrasound and a random zero sphygmomanometer placed just above the elbow. Ankle systolic pressure was measured in the posterior tibial artery of the right leg using a Doppler ultrasound and a random zero sphygmomanometer with the cuff position just above the malleolus. The ABI was derived by dividing the systolic blood pressure in the ankle to that in the arm. PAD was defined as having an ABI less than 0.90.

Demographic and medical information were obtained during a standardized interview. Participants were asked questions about their education, smoking status, and alcohol consumption. The MMSE (Folstein et al., 1975) was administered to screen for possible dementia. In addition, a medical history was taken, including diagnoses of hypertension, diabetes, cardiovascular disease, and cerebrovascular disease. Blood samples were taken for DNA extraction, and apolipoprotein E (*APOE*) genotyping was performed with TaqMan technology for single-nucleotide polymorphisms rs7412 and rs429358. In this study, *APOE* status was coded as ε4 or non-ε4 carrier. The participants also completed self-report questionnaires, including the Hospital Anxiety and Depression Scale (Zigmond & Snaith, 1983), from which the number of depressive symptoms was derived.

### Statistical Analyses

Demographic and health differences between the two groups (PAD vs. no PAD) were examined by χ^2^ (categorical variables) and *t* tests (continuous variables). The associations between ABI and cognitive performance were first analyzed according to diagnostic category (PAD vs. no PAD). Group differences in cognitive performance were analyzed with *t* tests. Thereafter, the ABI was treated as a continuous variable. Four regression models were fitted to the data. The most basic model included age and sex as covariates. The second model included age, sex, and age-11 IQ. In the third model, we excluded persons with ABI greater than 1.40 because these values fall outside of the normal range. Abnormally high values may be a sign of severe PAD because calcification of the artery walls may cause incompressible vessels (Aboyans et al., 2012). In the fourth model, we also excluded persons with a history of cardiovascular or cerebrovascular disease because we were interested in the effect of PAD in a normal elderly sample without a history of severe vascular disease. Finally, we examined possible interaction effects between *APOE* and ABI in the fourth model because the effect of vascular risk factors on cognition in some cases has been reported to be stronger among carriers of the *APOE* ε4 allele (Bender & Raz, 2012).

## Results

### LBC1921

The characteristics of the LBC1921 sample are shown in Table 1. By definition, persons with PAD had a lower ABI compared with persons without PAD. The PAD persons were also significantly more likely to be *APOE* ε4 carriers. However, there were no significant group differences for the demographic variables or childhood mental ability. Also, the correlation between childhood mental ability and ABI was not significant (*r* = .07, *p* = .41). As for the health-related variables, persons with PAD tended to be more affected by cardiovascular disease (*p* = .06). Differences in cognitive performance on the basis of the dichotomized variable (PAD vs. no PAD) are also reported in Table 1. There were no significant group differences, although the PAD group showed a marginally significant poorer performance on Digit Symbol (*p* = .05).[Table-anchor tbl1]

Table 2 shows the associations between ABI and cognitive performance. In these analyses, ABI was treated as a continuous variable. In Model 1 (controlling for age and sex), a higher ABI was significantly associated with better general cognitive ability (*g*), better working memory performance (Letter-Number Sequencing), and better performance in two of three processing speed tasks (Digit Symbol and mean simple RT). Controlling for childhood mental ability (Model 2) rendered the association between ABI and *g* nonsignificant and slightly weakened the associations with working memory and processing speed. After removing persons with abnormally high ABI (Model 3), general cognitive ability and Digit Symbol performance were significantly associated with ABI. Model 4 resulted in a similar pattern of results as Model 1, suggesting that in a sample free of severe vascular disease, higher ABI is associated with better general cognitive ability (*g* factor: B = 1.16, 95% confidence interval [CI] = 0.20–2.11) and processing speed (Digit Symbol: B = 19.73, 95% CI = 5.99–33.48; mean simple RT: B = −0.19, 95% CI = −0.34 to −0.04). Effect sizes expressed in *R*^2^ change, indicating the additional amount of variance explained by the ABI after having entered the covariates, ranged between .05 and .08 for the significant associations, with a larger effect observed for processing speed. Further adjustment for smoking status (never smoked vs. ex- or current smoker), alcohol consumption (no vs. yes), history of hypertension, or history of diabetes did not affect the pattern of results (data not shown). Controlling for the number of depressive symptoms did not attenuate the association between ABI and cognition. Rather, it resulted in slightly larger βs for ABI and rendered the associations with age-87 IQ (β = .18, *p* < .05) and Raven’s Matrices (β = .19, *p* < .05) significant. Finally, we checked for potential interaction effects between *APOE* and ABI. However, no such interaction effect was observed (data not shown).[Table-anchor tbl2]

### LBC1936

The characteristics of the LBC1936 sample are shown in Table 3. The prevalence of PAD was lower in the younger cohort (12% vs. 28% in LBC1921), consistent with previous findings that the prevalence of PAD increases with age (Aboyans & Criqui, 2009). Again, there were no group differences for the demographic variables or childhood mental ability. Also, there was no significant correlation between childhood mental ability and ABI (*r* = −.03, *p* = .45). As for the vascular risk factors, there was a higher prevalence of current smokers in the PAD group. Persons in this group also had higher systolic blood pressure and were more likely to have a history of diabetes and cardiovascular disease. Regarding the cognitive measures, there was a significant group difference in memory performance, with the PAD group showing better performance compared with the no PAD group.[Table-anchor tbl3]

Table 4 shows the associations between ABI and cognitive performance in the LBC1936. In Model 1 (controlling for age and sex), there was a significant negative association between ABI and Logical Memory total score. However, only this one test showed a significant association; furthermore, it was in the unexpected direction. The association with memory no longer remained significant after removing persons with a history of cardiovascular or cerebrovascular disease (Model 4). In Model 2 (controlling for age, sex, and age-11 IQ), there was a significant positive association between ABI and Digit Symbol (processing speed) performance, and in the fourth and final model, a significant association between ABI and cognitive performance was observed for Digit Symbol (B = 0.77, 95% CI = 0.21 to 1.34) and *g* speed (B = 8.60, 95% CI = 1.30 to 15.90). Effect sizes, expressed in *R*^2^ change, were .01, indicating that ABI explained 1% of the variance in processing speed in the younger cohort. Further adjustment for smoking status, alcohol consumption, history of hypertension, history of diabetes, or number of depressive symptoms did not affect the pattern of results. Also, no significant interaction effect between *APOE* and ABI was observed (data not shown).[Table-anchor tbl4]

## Discussion

In this study, we observed no significant group differences in cognitive performance between persons with or without PAD (ABI < 0.90). However, we did observe an association between a continuous measure of ABI and cognitive functioning. This association was most consistently found for processing speed.

One possible explanation for the association between ABI and cognitive performance would be that persons with poor mental abilities are at higher risk of developing PAD (Gale, Deary, Fowkes, & Batty, 2012). However, because we had data on childhood IQ, we were able to rule out that possibility and show that prior mental abilities could not account for this association. First, there was no significant difference in age-11 IQ between persons with or without PAD (Tables 1 and 3) or any correlation between childhood mental ability and ABI (*p*s > .40). Second, the association between ABI and cognitive performance was significant also after controlling for childhood mental ability (Tables 2 and 4). Thus, our results imply that PAD is associated with relative cognitive decline in older adults.

Another possible explanation for the association between ABI and cognitive performance would be a higher prevalence of severe vascular conditions in persons with low ABI. However, although persons with PAD tended to have higher prevalence of cardiovascular disease, excluding persons with self-reported cardiovascular or cerebrovascular disease did not result in weaker associations between ABI and cognition (Tables 2 and 4, Model 4). These results indicate that PAD is associated with impairment of cognitive function in relatively healthy older adults, free of stroke and symptomatic cardiovascular disease.

As indicated by the effect sizes, the association between ABI and cognition was more pronounced in the older cohort (87 years). This was not due to more severe PAD in the older cohort—mean ABI was very similar for the two groups with PAD. Furthermore, the prevalence of vascular disease was not higher in the old PAD group (Tables 1 and 3).

Why was the effect most evident in the older cohort? One explanation could be that the older persons with PAD had suffered from this condition for a longer time. The prevalence of PAD is strongly related to age (Aboyans & Criqui, 2009), which makes it more likely for the old cohort to have been affected by atherosclerosis during a more protracted period. Continued obstruction of the blood flow to the brain may lead to cerebral hypoperfusion, hampering efficient delivery of glucose and oxygen to the brain cells (de la Torre, 2000). Atherosclerotic disease has also been associated with increased number of silent brain infarcts (Bouchi et al., 2012; Longstreth et al., 2002) and white matter hyperintensities (Bos et al., 2011; Bots et al., 1993), both of which have been related to cognitive deficits (Wright et al., 2008). Thus, persons with PAD who are free of clinically manifest cerebrovascular disease could still have brain changes that may cause cognitive deficits (de la Torre, 2010; Rafnsson, Deary, & Fowkes, 2009).

An additional explanation for why the older cohort was more affected is that older age may be associated with increased vulnerability for different vascular conditions. Gray and white matter volumes are known to show age-related shrinkage, which is in turn related to poorer cognitive performance (Raz & Kennedy, 2009). Normal aging is also associated with reduced cerebral blood flow (de la Torre, 2000). Therefore, an older brain may be more likely to be affected by additional strain (e.g., reduced blood flow due to atherosclerotic disease). Taken together, these brain changes may lead to cognitive deficits and eventually dementia. In the younger cohort, there was a marginal effect of the ABI on the general cognitive factor, and in the older cohort this effect was significant. Thus, it is possible that continued exposure, especially in combination with age-related brain changes, will result in more global cognitive deficits.

For both cohorts, the strongest association was observed for processing speed. This is consistent with previous findings that persons affected by PAD perform worse on the Digit Symbol task (Haan et al., 1999; Price et al., 2006; Vupputuri et al., 2008). In the present study, a higher ABI was associated with better performance on Digit Symbol (higher score) and RT (faster performance) in the older cohort and better performance on Digit Symbol and *g* speed in the younger cohort. This is consistent with previous studies observing associations between cerebral blood flow, white matter hyperintensities, and silent infarcts with processing speed (Marquine et al., 2010; Rabbitt et al., 2006; Vermeer et al., 2003).

Major strengths of the present study are that we had access to two large age-homogenous population-based samples of different ages in which ABI data were collected in the same way by the same set of research nurses. Both cohorts were tested with broad cognitive test batteries, which were largely overlapping in terms of cognitive domains assessed and tests used, and we had access to childhood mental ability scores.

A limitation of the present study is that vascular disease history (e.g., stroke, cardiovascular disease) was measured by self-report, which is always associated with some uncertainty. It should also be mentioned that analyses were performed for a relatively large number of cognitive tests, thus increasing the risk of committing a Type I error. If we had applied a more stringent α level (e.g., *p* < .01), only the associations with processing speed would have remained significant. Another limitation is that although the samples were population based, they are still likely to be of superior health compared with the general population. Persons with severe health problems would have been more likely to decline participation in these quite extensive examinations. Moreover, because PAD is related to increased mortality (Criqui et al., 1992), the most severe cases might already be dead. This selectivity is most likely to affect the results by leading to an underestimation of the association between ABI and cognition.

The results of this study show that atherosclerosis, measured with the ABI, is associated with worse cognitive performance in a nondemented population-based sample, especially among the oldest old (>85 years). These findings are highly relevant given that vascular cognitive impairment is a common and possibly preventable condition that is associated with a high risk of progressing to dementia or dying (Wentzel et al., 2001). The ABI is an easily applied measure, which at a low cost can provide an assessment of PAD and generalized atherosclerosis without any risk for the patient. Given the association between ABI and cognition, the ABI could be a clinically useful tool to provide important information pertaining to vascular health for the oldest old who are at risk for cognitive decline. Early detection of persons at high risk enables intervention at an early stage, when it is most like to be efficient. Control of vascular risk factors could have a major effect on promoting healthy aging in the general population (de la Torre, 2010).

## Figures and Tables

**Table 1 tbl1:** Characteristics of the LBC1921 Sample (*n* = 170) According to PAD Status

Characteristic	No PAD *n* = 123		PAD *n =* 47		Effect Size
	*M*	*SD*	*M*	*SD*	
ABI	1.13	0.18	0.75	0.12	2.28**
Age	86.62	0.40	86.58	0.40	0.11
Sex (% women)	50.41		55.32		0.04
Education (years)	11.45	2.77	10.90	2.27	0.21
Age-11 IQ	103.43	14.04	101.90	11.78	0.11
MMSE	28.16	1.51	28.17	1.51	0.01
*APOE* ε4 (%)	18.03		33.33		0.16*
Smoking					0.08
Never smoked (%)	50.41		42.55		
Ex-smoker (%)	47.15		53.19		
Current smoker (%)	2.44		4.26		
Alcohol consumption (yes, %)	76.03		74.47		0.02
Units of alcohol/week	8.19	11.51	8.81	11.50	0.05
Systolic blood pressure	155.58	21.14	160.49	23.74	0.23
Diastolic blood pressure	76.05	10.54	73.19	11.24	0.27
History of hypertension (%)	44.72		55.32		0.10
History of diabetes (%)	4.88		4.26		0.01
History of cardiovascular disease (%)	20.33		34.04		0.14
History of cerebrovascular disease (%)	8.13		6.38		0.03
Number of depressive symptoms	3.92	2.49	3.51	2.24	0.17
Age-87 IQ	101.24	13.75	100.42	14.29	0.06
*g* factor	0.11	0.94	–0.09	0.98	0.22
Raven’s Matrices	28.99	8.84	26.80	9.69	0.24
Logical Memory–total	34.26	13.30	31.77	15.26	0.18
Letter fluency	40.20	12.44	41.04	11.66	0.07
Letter-Number Sequencing	9.30	2.93	8.94	3.11	0.12
Digit Symbol	42.19	12.68	37.74	13.06	0.35
Mean simple RT	0.34	0.10	0.39	0.19	0.37
Mean four-choice RT (correct responses)	0.82	0.13	0.83	0.18	0.05
*Note*. For continuous variables, *p* values for group differences are based on *t* tests and absolute effect size is expressed as Cohen’s *d*. For categorical variables, *p* values are based on χ^2^ tests and effect size is expressed as ϕ/Cramer’s V.					
* *p* < .05. ** *p* < .001.					

**Table 2 tbl2:** Associations Between ABI and Cognitive Performance in the 1921 Cohort

Cognitive Variable	Model 1^a^			Model 2^b^		Model 3^c^		Model 4^d^		
	β	*p*	*R*^2^ Change	β	*p*	β	*p*	β	*p*	*R*^2^ Change
Age-87 IQ	.07	.38	.005	−.02	.81	.03	.72	.13	.17	.016
*g* Factor	.16	.04*	.025	.07	.37	.17	.04*	.23	.02*	.053
Raven’s Matrices	.11	.16	.012	.02	.84	.09	.28	.15	.12	.022
Logical Memory–total	.10	.18	.011	.05	.57	.11	.19	.19	.08	.034
Letter fluency	.09	.24	.008	.04	.65	.09	.27	.16	.11	.026
Letter-Number Sequencing	.22	<.01**	.047	.17	.04*	.11	.18	.18	.09	.031
Digit Symbol	.20	.01*	.039	.17	.04*	.25	<.01**	.29	<.01**	.084
Mean simple RT	−.16	.04*	.027	−.15	.08	−.17	.05	−.27	.01*	.071
Mean four-choice RT (correct responses)	−.05	.55	.002	.00	.97	−.04	.67	−.07	.55	.004
^a^ Adjusted for age and sex, *n* = 170. ^b^ Adjusted for age, sex, and age-11 IQ, *n* = 149. ^c^ Adjusted for age, sex, and age-11 IQ, persons with ABI > 1.40 excluded, *n* = 139. ^d^ Adjusted for age, sex, and age-11 IQ, persons with ABI > 1.40 or a history of cardiovascular or cerebrovascular disease excluded, *n* = 93.										
* *p* < .05. ** *p* < .01.										

**Table 3 tbl3:** Characteristics of the LBC1936 Sample (*n* = 748) According to PAD Status

Characteristics	No PAD *n* = 657		PAD *n* = 91		Effect Size
	*M*	*SD*	*M*	*SD*	
ABI	1.12	0.16	0.81	0.09	2.06***
Age	72.53	0.70	72.47	0.68	0.09
Sex (% women)	48.86		48.35		0.00
Education (years)	10.82	1.15	10.79	1.10	0.03
Age-11 IQ	101.44	14.72	101.56	13.62	0.01
MMSE	28.84	1.29	28.75	1.30	0.07
*APOE* ε4 (%)	30.39		27.06		0.02
Smoking					0.12**
Never smoked (%)	48.55		39.56		
Ex-smoker (%)	43.99		42.86		
Current smoker (%)	7.46		17.58		0.12**
Alcohol consumption (yes, %)	89.04		82.42		0.07
Units of alcohol/week	13.24	14.81	14.98	12.89	0.12
Systolic blood pressure	146.81	18.36	154.39	18.54	0.41***
Diastolic blood pressure	77.65	9.49	78.73	10.84	0.11
History of hypertension (%)	48.10		53.85		0.04
History of diabetes (%)	8.68		16.48		0.09*
History of cardiovascular disease (%)	27.25		38.46		0.08*
History of cerebrovascular disease (%)	5.48		8.79		0.05
Number of depressive symptoms	2.52	2.16	2.86	2.20	0.16
*g* Factor	0.04	0.96	0.11	0.98	0.07
*g* Memory	0.01	0.97	0.29	0.96	0.29
*g* Speed	0.05	0.98	−0.03	1.01	0.08
Matrix Reasoning	13.26	4.92	14.10	5.01	0.17
Logical Memory–total	45.67	9.83	49.10	10.35	0.35**
Letter fluency	43.34	12.97	45.15	13.50	0.14
Letter-Number Sequencing	10.94	3.03	11.60	3.01	0.22
Digit Symbol	57.00	12.23	55.91	12.21	0.09
Mean simple RT	0.27	0.05	0.27	0.04	0.15
Mean four-choice RT (correct responses)	0.65	0.09	0.64	0.09	0.10
*Note*. For continuous variables, *p* values for group differences are based on *t* tests and absolute effect size is expressed as Cohen’s *d*. For categorical variables, *p* values are based on χ^2^ tests and effect size is expressed as ϕ/Cramer’s V.					
* *p* < .05. ** *p* < .01. *** *p* < .001.					

**Table 4 tbl4:** Associations Between ABI and Cognitive Performance in the 1936 Cohort

Cognitive Variable	Model 1^a^			Model 2^b^		Model 3^c^		Model 4^d^		
	β	*p*	*R*^2^ Change	β	*p*	β	*p*	β	*p*	*R*^2^ Change
*g* Factor	.01	.72	.000	.05	.12	.04	.21	.07	.07	.005
*g* Memory	−.05	.20	.002	−.02	.48	−.05	.15	.00	.95	.000
*g* Speed	.04	.35	.001	.06	.09	.06	.10	.12	<.01**	.014
Matrix Reasoning	−.01	.72	.000	.01	.74	.01	.77	.03	.47	.001
Logical Memory–total	−.09	.01*	.008	−.07	.04*	−.08	.03*	−.04	.33	.002
Letter fluency	.01	.81	.000	.03	.34	.02	.59	.04	.40	.001
Letter-Number Sequencing	−.01	.70	.000	.02	.62	.01	.87	.04	.38	.001
Digit Symbol	.06	.11	.003	.09	.01*	.06	.07	.10	.02*	.009
Mean simple RT	−.01	.74	.000	−.03	.47	−.02	.58	−.08	.11	.006
Mean four-choice RT (correct responses)	.01	.81	.000	−.01	.76	−.00	.99	−.04	.42	.001
^a^ Adjusted for age and sex, *n* = 748. ^b^ Adjusted for age, sex, and age-11 IQ, *n* = 706. ^c^ Adjusted for age, sex, and age-11 IQ, persons with ABI > 1.40 excluded, *n* = 673. ^d^ Adjusted for age, sex, and age-11 IQ, persons with ABI > 1.40 or a history of cardiovascular or cerebrovascular disease excluded, *n* = 455.										
* *p* < .05. ** *p* < .01.										

## References

[c1] Aboyans V., & Criqui M. H. (2009). The epidemiology of peripheral arterial disease In Robert D. (Ed.), Peripheral arterial disease (pp. 1–25). New York, NY: McGraw-Hill

[c2] Aboyans V., Criqui M. H., Abraham P., Allison M. A., Creager M. A., Diehm C., . . . Treat-Jacobson D. (2012). Measurement and interpretation of the Ankle-Brachial Index. A scientific statement from the American Heart Association. Circulation, 126, 2890–2909. doi:10.1161/CIR.0b013e318276fbcb 23159553

[c3] Bender A. R., & Raz N. (2012). Age-related differences in episodic memory: A synergistic contribution of genetic and physiological vascular risk factors. Neuropsychology, 26, 442–450. doi:10.1037/a0028669 22612575

[c4] Bos D., Ikram M. A., Elias-Smale S. E., Krestin G. P., Hofman A., Witteman J. C. M., . . . Vernooij M. W. (2011). Calcification in major vessel beds relates to vascular brain disease. Arteriosclerosis and Thrombosis: A Journal of Vascular Biology, 31, 2331–2337. doi:10.1161/ATVBAHA.111.232728 21868705

[c5] Bots M. L., van Swieten J. C., Breteler M. M., de Jong P. T., van Gijn J., Hofman A., & Grobbee D. E. (1993). Cerebral white matter lesions and atherosclerosis in the Rotterdam Study. Lancet, 341, 1232–1237. doi:10.1016/0140-6736(93)91144-B 8098390

[c6] Bouchi R., Babazono T., Takagi M., Yoshida N., Nyumura I., Toya K., . . . Uchigata Y. (2012). Non-linear association between ankle-brachial pressure index and prevalence of silent cerebral infarction in Japanese patients with type 2 diabetes. Atherosclerosis, 222, 490–494. doi:10.1016/j.atherosclerosis.2012.02.025 22460047

[c7] Bowler J. V., Steenhuis R., & Hachinski V. (1999). Conceptual background to vascular cognitive impairment. Alzheimer Disease and Associated Disorders, 13, S30–37 10609679

[c8] Corley J., Jia X., Kyle A. M., Gow A. J., Brett C. E., Starr J. M., . . . Deary I. J. (2010). Caffeine consumption and cognitive function at age 70: The Lothian Birth Cohort 1936 study. Psychosomatic Medicine, 72, 206–214. doi:10.1097/PSY.0b013e3181c92a9c 19995882

[c9] Criqui M. H., Langer R. D., Fronek A., Feigelson H. S., Klauber M. R., McCann T. J., & Browner D. (1992). Mortality over a period of 10 years in patients with peripheral arterial disease. New England Journal of Medicine, 326, 381–386. doi:10.1056/NEJM199202063260605 1729621

[c10] Deary I. J., Der G., & Ford G. (2001). Reaction times and intelligence differences: A population-based cohort study. Intelligence, 29, 389–399. doi:10.1016/S0160-2896(01)00062-9

[c11] Deary I. J., Gow A. J., Pattie A., & Starr J. M. (2012). Cohort profile: The Lothian Birth Cohorts of 1921 and 1936. International Journal of Epidemiology, 41, 1576–1584. doi:10.1093/ije/dyr197 22253310

[c12] Deary I. J., Gow A. J., Taylor M. D., Corley J., Brett C., Wilson V., & Starr J. M. (2007). The Lothian Birth Cohort 1936: A study to examine influences on cognitive ageing from age 11 to age 70 and beyond. BMC Geriatrics, 7, 28. doi:10.1186/1471-2318-7-28 18053258 PMC2222601

[c13] Deary I. J., Simonotto E., Meyer M., Marshall A., Marshall I., Goddard N., & Wardlaw J. M. (2004a). The functional anatomy of inspection time: An event-related fMRI study. NeuroImage, 22, 1466–1479. doi:10.1016/j.neuroimage.2004.03.047 15275904

[c14] Deary I. J., Whalley L. J., Lemmon H., Crawford J. R., & Starr J. M. (2000). The stability of individual differences in mental ability from childhood to old age: Follow-up of the 1932 Scottish Mental Survey. Intelligence, 28, 49–55. doi:10.1016/S0160-2896(99)00031-8

[c15] Deary I. J., Whiteman M. C., Starr J. M., Whalley L. J., & Fox H. C. (2004b). The impact of childhood intelligence on later life: Following up the Scottish Mental Surveys of 1932 and 1947. Journal of Personality and Social Psychology, 86, 130–147. doi:10.1037/0022-3514.86.1.130 14717632

[c16] de la Torre J. C. (2000). Critically attained threshold of cerebral hypoperfusion: The CATCH hypothesis of Alzheimer’s pathogenesis. Neurobiology of Aging, 21, 331–342. doi:10.1016/S0197-4580(00)00111-1 10867218

[c17] de la Torre J. C. (2009). Cerebrovascular and cardiovascular pathology in Alzheimer’s disease. International Review of Neurobiology, 84, 35–48. doi:10.1016/S0074-7742(09)00403-6 19501712

[c18] de la Torre J. C. (2010). Vascular risk factor detection and control may prevent Alzheimer’s disease. Ageing Research Reviews, 9, 218–225. doi:10.1016/j.arr.2010.04.002 20385255

[c19] Folstein M. F., Folstein S. E., & McHugh P. R. (1975). Mini-mental state. A practical method for grading the cognitive state of patients for the clinician. Journal of Psychiatric Research, 12, 189–198. doi:10.1016/0022-3956(75)90026-6 1202204

[c20] Fowkes F. G. R. (1988). The measurement of atherosclerotic peripheral arterial disease in epidemiological surveys. International Journal of Epidemiology, 17, 248–254. doi:10.1093/ije/17.2.248 3042648

[c21] Fowkes F. G. R., Murray G. D., Butcher I., Heald C. L., Lee R. J., Chamless L. E., . . . McDermott M. M. Ankle Brachial Index Collaboration (2008). Ankle brachial Index combined with Framingham Risk Score to predict cardiovascular events and mortality: A meta-analysis. Journal of the American Medical Association, 300, 197–208. doi:10.1001/jama.300.2.197 18612117 PMC2932628

[c22] Gale C. R., Deary I. J., Fowkes F. G., & Batty G. D. (2012). Intelligence in early adulthood and subclinical atherosclerosis in middle-aged men: The Vietnam Experience Study. Journal of Epidemiology and Community Health, 66, e13. doi:10.1136/jech.2010.109835 21558481

[c23] Guerchet M., Aboyans V., Nubukpo P., Lacroix P., Clément J.-P., & Preux P.-M. (2011). Ankle-brachial index as a marker of cognitive impairment and dementia in general population. a systematic review. Atherosclerosis, 216, 251–257. doi:10.1016/j.atherosclerosis.2011.03.024 21497350

[c24] Haan M. N., Shemanski L., Jagust W. J., Manolio T. A., & Kuller L. (1999). The role of APOE ε4 in modulating effects of other risk factors for cognitive decline in elderly persons. JAMA: Journal of the American Medical Association, 282, 40–46. doi:10.1001/jama.282.1.40 10404910

[c25] Hart C. L., Taylor M. D., Davey Smith G., Whalley L. J., Starr J. M., Hole D. J., . . . Deary I. J. (2004). Childhood IQ and cardiovascular disease in adulthood: Prospective observational study linking the Scottish Mental Survey 1932 and the Midspan studies. Social Science & Medicine, 59, 2131–2138. doi:10.1016/j.socscimed.2004.03.016 15351478

[c26] Jellinger K. A. (2008). The pathology of “vascular dementia”: a critical update. Journal of Alzheimer’s Disease, 14, 107–123 10.3233/jad-2008-14110 18525132

[c27] Johnson W., Price J. F., Rafnsson S. B., Deary I. J., & Fowkes F. G. R. (2010). Ankle-brachial index predicts level of, but not change in, cognitive function: The Edinburgh Artery Study at the 15-year follow-up. Vascular Medicine, 15, 91–97. doi:10.1177/1358863X09356321 20147579

[c28] Lezak M. (2004). Neuropsychological testing. Oxford, United Kingdom: Oxford University Press

[c29] Longstreth W. T., Dulberg C., Manolio T. A., Lewis M. R., Beachamp N. J., O’Leary D., . . . Furberg C. D. (2002). Incidence, manifestations, and predictors of brain infarcts defined by serial cranial magnetic resonance imaging in the elderly: The Cardiovascular Health Study. Stroke, 33, 2376–2382. doi:10.1161/01.STR.0000032241.58727.49 12364724

[c30] Luciano M., Gow A. J., Harris S. E., Hayward C., Allerhand M., Starr J. M., . . . Deary I. J. (2009). Cognitive ability at age 11 and 70 years, information processing speed, and APOE variation: The Lothian Birth Cohort 1936 study. Psychology and Aging, 24, 129–138. doi:10.1037/a0014780 19290744

[c31] Marquine M. J., Attix D. K., Goldstein L. B., Samsa G. P., Payne M. E., Chelune G. J., & Steffens D. C. (2010). Differential patterns of cognitive decline in anterior and posterior white matter hyperintensity progression. Stroke, 41, 1946–1950. doi:10.1161/STROKEAHA.110.587717 20651266 PMC3279727

[c32] McGurn B., Deary I. J., & Starr J. M. (2008). Childhood cognitive ability and risk of late-onset Alzheimer and vascular dementia. Neurology, 71, 1051–1056. doi:10.1212/01.wnl.0000319692.20283.10 18579804

[c33] Murphy T. P., Dhangana R., Pencina M. J., & D’Agostino R. B. (2012). Ankle-brachial index and cardiovascular risk prediction: An analysis of 11,594 individuals with 10-year follow-up. Atherosclerosis, 220, 160–167. doi:10.1016/j.atherosclerosis.2011.10.037 22099055

[c34] O’Brien J. T. (2006). Vascular cognitive impairment. The American Journal of Geriatric Psychiatry, 14, 724–733. doi:10.1097/01.JGP.0000231780.44684.7e 16943169

[c35] Price J. F., McDowell S., Whiteman M. C., Deary I. J., Stewart M. C., & Fowkes F. G. R. (2006). Ankle brachial index as a predictor of cognitive impairment in the general population: Ten-year follow-up of the Edinburgh Artery Study. Journal of the American Geriatrics Society, 54, 763–769. doi:10.1111/j.1532-5415.2006.00702.x 16696741

[c36] Rabbitt P., Scott M., Thacker N., Lowe C., Jackson A., Horan M., & Pendelton N. (2006). Losses in gross brain volume and cerebral blood flow account for age-related differences in speed but not in fluid intelligence. Neuropsychology, 20, 549–557. doi:10.1037/0894-4105.20.5.549 16938017

[c37] Rafnsson S. B., Deary I. J., & Fowkes F. G. (2009). Peripheral arterial disease and cognitive function. Vascular Medicine, 14, 51–61. doi:10.1177/1358863X08095027 19144780

[c38] Rafnsson S. B., Deary I. J., Smith F. B., Whiteman M. C., & Fowkes F. G. R. (2007). Cardiovascular diseases and decline in cognitive functioning in an elderly community population: The Edinburgh Artery Study. Psychosomatic Medicine, 69, 425–434. doi:10.1097/psy.0b013e318068fce4 17556639

[c39] Raven J. C., Court J. H., & Raven J. (1977). Manual for Raven’s Progressive Matrices and Vocabulary Scales. London, United Kingdom: H. K. Lewis

[c40] Raz N., & Kennedy K. M. (2009). A systems approach to the aging brain: Neuroanatomic changes, their modifiers, and cognitive correlates In Jagust W. & D’Esposito M. (Eds.), Imaging the aging brain (pp. 43–70). New York, NY: Oxford University Press. doi:10.1093/acprof:oso/9780195328875.003.0004

[c41] Reitz C., Tang M. X., Schupf N., Manly J. J., Mayeux R., & Luchsinger J. A. (2010). A summary risk score for the prediction of Alzheimer disease in elderly persons. Archives of Neurology, 67, 835–841. doi:10.1001/archneurol.2010.136 20625090 PMC3068839

[c42] Resnick H. E., Lindsay R. S., McDermott M. M., Devereux R. B., Jones K. L., Fabsitz R. R., & Howard B. V. (2004). Relationship of high and low ankle brachial index to all-cause and cardiovascular disease mortality: The Strong Heart Study. Circulation, 109, 733–739. doi:10.1161/01.CIR.0000112642.63927.54 14970108

[c43] Rockwood K., Moorhouse P. K., Song X., MacKnight C., Gauthier S., Kertesz A., . . . Feldman H. (2007). Disease progression in vascular cognitive impairment: Cognitive, functional and behavioral outcomes in the Consortium to Investigate Vascular Impairment of Cognition (CIVIC) cohort study. Journal of the Neurological Sciences, 252, 106–112. doi:10.1016/j.jns.2006.10.015 17189642

[c44] Rockwood K., Wentzel C., Hachinski V., Hogan D. B., MacKnight C., & McDowell I. (2000). Prevalence and outcomes of vascular cognitive impairment. Vascular Cognitive Impairment Investigators of the Canadian Study of Health and Aging. Neurology, 54, 447–451. doi:10.1212/WNL.54.2.447 10668712

[c45] Scottish Council for Research in Education (1933). The intelligence of Scottish children: A national survey of an age-group. London, United Kingdom: University of London Press

[c46] Scottish Council for Research in Education (1949). The intelligence of Scottish children: A comparison on the 1947 and 1932 surveys of the intelligence of eleven-year-old pupils. London, United Kingdom: University of London Press

[c47] Starr J. M., Taylor M. D., Hart C., Davey Smith G., Whalley L. J., Hole D. J., . . . Deary I. J. (2004). Childhood mental ability and blood pressure at midlife: Linking the Scottish Mental Survey 1932 and the Midspan studies. Journal of Hypertension, 22, 893–897. doi:10.1097/00004872-200405000-00009 15097227

[c48] Vermeer S. E., Prins N. D., den Heijer T., Hofman A., Koudstaal P. J., & Breteler M. M. (2003). Silent brain infarcts and the risk of dementia and cognitive decline. The New England Journal of Medicine, 348, 1215–1222. doi:10.1056/NEJMoa022066 12660385

[c49] Vupputuri S., Shoham D. A., Hogan S. L., & Kshirsagar A. V. (2008). Microalbuminuria, peripheral artery disease, and cognitive function. Kidney International, 73, 341–346. doi:10.1038/sj.ki.5002672 18033244

[c50] Waldstein S. R., Tankard C. F., Maijer K. J., Pelletier J. R., Snow J., Gardner A. W., . . . Katzel L. I. (2003). Peripheral arterial disease and cognitive function. Psychosomatic Medicine, 65, 757–763. doi:10.1097/01.PSY.0000088581.09495.5E 14508017

[c51] Wechsler D. (1987). Wechsler Memory Scale—Revised. New York, NY: Psychological Corporation

[c52] Wechsler D. (1998a). WAIS-III^*UK*^ administration and scoring manual. London, United Kingdom: Psychological Corporation

[c53] Wechsler D. (1998b). WMS-III^*UK*^ administration and scoring manual. London, United Kingdom: Psychological Corporation

[c54] Wentzel C., Rockwood K., MacKnight C., Hachinski V., Hogan D. B., Feldman H., . . . McDowell I. (2001). Progression of impairment in patients with vascular cognitive impairment without dementia. Neurology, 57, 714–716. doi:10.1212/WNL.57.4.714 11524488

[c55] Wright C. B., Festa J. R., Paik M. C., Schmiedigen A., Brown T. R., Yoshita M., . . . Stern Y. (2008). White matter hyperintensities and subclinical infarction: Associations with psychomotor speed and cognitive flexibility. Stroke, 39, 800–805. doi:10.1161/STROKEAHA.107.484147 18258844 PMC2267752

[c56] Zigmond A. S., & Snaith R. P. (1983). The Hospital Anxiety and Depression Scale. Acta Psychiatrica Scandinavica, 67, 361–370. doi:10.1111/j.1600-0447.1983.tb09716.x 6880820

